# Gene expression profiles from discordant monozygotic twins suggest that molecular pathways are shared among multiple systemic autoimmune diseases

**DOI:** 10.1186/ar3330

**Published:** 2011-04-26

**Authors:** Terrance P O'Hanlon, Lisa G Rider, Lu Gan, Rick Fannin, Richard S Paules, David M Umbach, Clarice R Weinberg, Ruchir R Shah, Deepak Mav, Mark F Gourley, Frederick W Miller

**Affiliations:** 1 Environmental Autoimmunity Group, National Institute of Environmental Health Sciences, National Institutes of Health, 9000 Rockville Pike, Bethesda, MD 20892, USA; 2 Laboratory of Toxicology and Pharmacology, National Institute of Environmental Health Sciences, National Institutes of Health, 111 T.W. Alexander Drive, Research Triangle Park, NC 27709, USA; 3 Biostatistics Branch, National Institute of Environmental Health Sciences, National Institutes of Health, 111 T.W. Alexander Drive, Research Triangle Park, NC 27709, USA; 4 SRA International Inc., 2605 Meridian Parkway, Durham, NC 27713, USA; 5 National Institute of Arthritis and Musculoskeletal Disease, National Institutes of Health, 9000 Rockville Pike, Bethesda, MD 20892, USA

## Abstract

**Introduction:**

The objective of this study is to determine if multiple systemic autoimmune diseases (SAID) share gene expression pathways that could provide insights into pathogenic mechanisms common to these disorders.

**Methods:**

RNA microarray analyses (Agilent Human 1A(V2) 20K oligo arrays) were used to quantify gene expression in peripheral blood cells from 20 monozygotic (MZ) twin pairs discordant for SAID. Six affected probands with systemic lupus erythematosus (SLE), six with rheumatoid arthritis (RA), eight with idiopathic inflammatory myopathies (IIM), and their same-gendered unaffected twins, were enrolled. Comparisons were made between discordant twin pairs and these were also each compared to 40 unrelated control subjects (matched 2:1 to each twin by age, gender and ethnicity) using statistical and molecular pathway analyses. Relative quantitative PCR was used to verify independently measures of differential gene expression assessed by microarray analysis.

**Results:**

Probands and unrelated, matched controls differed significantly in gene expression for 104 probes corresponding to 92 identifiable genes (multiple-comparison adjusted *P *values < 0.1). Differentially expressed genes involved several overlapping pathways including immune responses (16%), signaling pathways (24%), transcription/translation regulators (26%), and metabolic functions (15%). Interferon (IFN)-response genes (*IFI27*, *OASF*, *PLSCR1*, *EIF2AK2*, *TNFAIP6*, and *TNFSF10*) were up-regulated in probands compared to unrelated controls. Many of the abnormally expressed genes played regulatory roles in multiple cellular pathways. We did not detect any probes expressed differentially in comparisons among the three SAID phenotypes. Similarly, we found no significant differences in gene expression when comparing probands to unaffected twins or unaffected twins to unrelated controls. Gene expression levels for unaffected twins appeared intermediate between that of probands and unrelated controls for 6535 probes (32% of the total probes) as would be expected by chance. By contrast, in unaffected twins intermediate ordering was observed for 84 of the 104 probes (81%) whose expression differed significantly between probands and unrelated controls.

**Conclusions:**

Alterations in expression of a limited number of genes may influence the dysregulation of numerous, integrated immune response, cell signaling and regulatory pathways that are common to a number of SAID. Gene expression profiles in peripheral blood suggest that for genes in these critical pathways, unaffected twins may be in a transitional or intermediate state of immune dysregulation between twins with SAID and unrelated controls, perhaps predisposing them to the development of SAID given the necessary and sufficient environmental exposures.

## Introduction

Previous studies suggest that patterns of gene expression as measured by RNA microarray are correlated among healthy twins, siblings, and other first-degree family members [1-3]. These data support the importance of shared genetic and perhaps environmental influences on transcription and other controls of steady-state mRNA expression. Interestingly, similar profiles of global RNA expression are observed among first-degree family members discordant for autoimmune disease, suggesting that other healthy, first-degree relatives may have an underlying genetic predisposition to disease [2,4,5].

Shared patterns of global gene expression have also been observed among genetically unrelated individuals with systemic autoimmune diseases (SAIDs), including systemic lupus erythematosus (SLE) and rheumatoid arthritis (RA) [6-9]. These data support the hypothesis that different autoimmune diseases share certain clinical features and perhaps mechanisms of disease pathogenesis. Most notably, the type I IFN 'signature' associated with the transcriptional control of many IFN-regulated gene products has been consistently detected among patients with SLE, dermatomyositis (DM), or other SAIDs [8,10-16]. Conversely, disease-specific gene expression profiles are associated with particular autoimmune pathologies and may reflect target tissue specificity and other distinctions in genetic susceptibilities or environmental exposure histories or both [7,17-20].

To minimize the role of polymorphic gene effects, we enrolled 20 pairs of monozygotic (MZ) twins discordant for SAID (six probands with SLE, six with RA, and eight with idiopathic inflammatory myopathies, or IIMs) and used RNA microarrays (Agilent Human 1A(V2); 20K) to examine gene expression patterns in their peripheral blood cells. To determine whether unaffected twins might show preclinical evidence of enhanced susceptibility to SAID, we also compared probands and their unaffected twins with 40 unrelated, matched control subjects. One might expect etiologically relevant genes to show expression patterns in the unaffected twins that are intermediate between those in their affected twin counterparts and in unrelated, matched controls. Moreover, in an effort to identify shared molecular pathways involved in the pathogenesis of these SAIDs, we asked whether particular gene expression profiles were characteristic of affected probands regardless of specific disease diagnosis.

## Materials and methods

### Study subjects

Five adult (at least 18 years of age) and 15 juvenile MZ twin pairs discordant for SAID and 40 unrelated control subjects (two controls per twin pair) matched on age within 6 years, gender, and ethnicity were subjects in this study. These subjects were among those enrolled between 2001 and 2006 in the National Institutes of Health (NIH) investigational review board-approved Twins-Sib study assessing the pathogenesis of SAID. Ethical approval for this microarray study was also obtained from the NIH investigational review board, and all human subjects provided informed consent. Twin pairs enrolled within 4 years of probands' diagnoses included 19 non-Hispanic Caucasian twin pairs and a single Hispanic twin pair (with SLE). Probands fulfilled American College of Rheumatology criteria for adult or juvenile SLE (*n *= 4 and 2, respectively), RA or JRA (*n *= 1 and 5, respectively), juvenile dermatomyositis (JDM) (*n *= 7), or juvenile polymyositis (JPM) (*n *= 1); we excluded patients with inherited, metabolic, infectious, or other causes of disease. The juvenile probands ranged in age from 3 to 18 years (mean of 11.2 years), whereas adults ranged from 19 to 43 years (mean of 29.2 years). Twins included 14 female and 6 male pairs. Monozygosity was confirmed by short tandem repeat analysis of genomic DNAs (Proactive Genetics, Inc., Augusta, GA, USA). Unrelated, matched controls were free of infections, trauma, vaccines, and surgeries for 8 weeks and had no first-degree family members with SAID.

RNA was purified from peripheral whole blood samples collected in PAXgene RNA tubes (VWR Scientific, Radnor, PA, USA) by using a PAXgene RNA Isolation Kit (Qiagen, Inc., Valencia, CA, USA) in accordance with the recommendations of the manufacturer. Total RNA was quantified spectrophotometrically and then stored at -80°C until analysis. To minimize sources of variability, we collected whole blood samples in the morning, and immunosuppressive therapy was held at least 24 hours before collection.

### Microarray analysis

Gene expression analysis was conducted by using Agilent Human 1A(V2) Oligo arrays with approximately 20,000 probes for known genes and expressed sequence tags (Agilent Technologies, Inc., Santa Clara, CA, USA). Each sample was hybridized against a human universal RNA control (Stratagene, La Jolla, CA, USA). Total RNA (500 ng) was amplified and labeled by using the Agilent Low RNA Input Fluorescent Linear Amplification Kit in accordance with the protocol of the manufacturer. For each two-color comparison, 750 ng of each Cy3- (universal control) and Cy5-labeled sample cRNA was mixed and fragmented by using the Agilent *in situ *Hybridization Kit. Hybridizations were performed for 17 hours in a rotating hybridization oven. Slides were washed and then scanned with an Agilent scanner. Data were obtained by using Agilent Feature Extraction software (version 7.5), with defaults for all parameters. The Agilent Feature Extraction Software performed error modeling, adjusting for additive and multiplicative noise. The resulting data were processed by using the Rosetta Resolver system (version 7.2) (Rosetta Biosoftware, now part of Microsoft Corporation, Redmond, WA, USA). Resulting base 2 logarithms of expression ratios (subject over universal control) were exported from Rosetta Resolver for further analysis. The data reported in this publication have been deposited in the National Center for Biotechnology Information's Gene Expression Omnibus (GEO) [21] and are accessible through GEO Series accession number [GEO:GSE24060].

### Microarray data normalization and quality control

We used principal component analysis (PCA) to investigate the effect of known sample variables such as subject age, gender, race, disease status, disease activity, medications, and peripheral white blood cell counts and differentials as well as technical variables such as sample hybridizations performed at different times. We observed no obvious differences across the first three principal components due to age, gender, ethnicity, disease status, disease activity, or treatment. We noticed, however, that samples clustered according to their hybridization batch. To correct for this, we centered the ratios for each probe by using the median for their hybridization group. The PCA plots generated by using the corrected data showed no obvious groupings, so we used these data for further analyses.

### Identification of differentially expressed probes

To identify probes that were differentially expressed in probands (P) as compared with their unaffected twin (U) and with unrelated, matched controls (C), we employed a mixed-effects model to calculate *P *values for each of the approximately 20,000 probes. Random effects included indicators for twin pairs, for P-versus-U comparisons, and for the strata defined by the matching variables for the P-versus-C and U-versus-C comparisons. With these adjustments, we performed three binary comparisons: (a) P versus C, in which all probands with SAID were compared with two unrelated, matched controls; (b) U versus C, in which all unaffected twins were compared with two unrelated, matched controls; (c) P versus U, in which all probands with SAID were compared with their unaffected twins in a paired analysis. We adjusted *P *values from each of the above three comparisons to control the false discovery rate (FDR) at 0.1 by using the Benjamini-Hochberg method [22].

### Disease-specific gene expression analyses

The goal was to investigate whether blood cell gene expression profiles for probands with SAID - namely (J)RA, (J)SLE, and (J)IIM (that is, JDM + JPM) - show characteristic differences in gene expression. To answer this question, we used all probe expression data and employed three different types of statistical analyses: (a) Hierarchical clustering - we performed unsupervised hierarchical clustering by using all 20 proband samples to check whether probands cluster by their known SAID subtype. (b) Multiclass analysis of variance (ANOVA) - to assess whether the difference between the gene expression of a proband and their respective twin is SAID subtype-specific, we first obtained a measurement of the gene expression difference per twin pair for each probe by subtracting the log-transformed expression value of the unaffected twin from that of the respective affected twin. We next grouped twin pairs into the three major SAID diagnostic classes. Finally, we performed a three-category, one-way ANOVA by using the differences in log-transformed expression values for each oligo probe. The *P *values were then adjusted for multiple testing by using the Benjamini-Hochberg method with the FDR of 0.1 [22]. (c) Mixed-effects model - to assess whether the difference between the gene expression of an affected proband and their corresponding unrelated but matched controls is SAID subtype-specific, we employed a mixed-effects model allowing a random effect for each matched set and treating the disease subtype as a fixed effect. The *P *values were then adjusted for multiple testing by using the Benjamini-Hochberg method with the FDR of 0.1 [22].

### Functional analysis of differentially expressed genes

We used Ingenuity Pathways Analysis (IPA) software (Ingenuity^® ^Systems, Redwood City, CA, USA) to assess functional class membership for probes identified as differentially expressed between probands and unrelated, matched controls. Each gene of interest was mapped and overlaid onto a global molecular network developed from information contained in the IPA Knowledge Base. Networks of genes were then generated algorithmically on the basis of their connectivity as established in the published literature. Fisher's exact test was used to assess whether the number of probes mapped to each biologic function or pathway (or both) differs from the number expected due to chance alone.

### Real-time polymerase chain reaction analysis

Relative quantitation (RQ) measurements of gene expression were by real-time polymerase chain reaction (RT-PCR) assays using cDNA prepared from Paxgene-purified whole peripheral blood RNA preparations collected from disease-discordant twins and unrelated, matched controls as described above. cDNA was prepared by using a High Capacity cDNA Reverse Transcription kit (1.0 μg of input RNA per subject) in accordance with the recommendations of the manufacturer (Applied Biosystems, Foster City, CA, USA). RT-PCRs were performed in triplicate by using commercially prepared cDNA primers and probes (TaqMan Gene Expression Assays; Applied Biosystems) for the following genes of interest: *MAP2K6 *(TaqMan Assay Hs00992389), *IL1RN *(Hs00893626), *IFI27 *(Hs00271467), *FCER1A *(Hs01090134), *FYN *(Hs00176628), *LTK *(Hs00914334), *KRTCAP2 *(Hs00744717), *SYTL2 *(Hs00262988), *ANXA3 *(Hs00971411), *CEACAM6 *(Hs00366002), *DEFA4 *(Hs00157252), *TNFAIP6 *(Hs01113602), *TNFSF10 *(Hs00234356), *EIF2AK2 *(Hs00169345), and *LGR6 *(Hs00663887). Genes of interest were selected from the microarray analysis by a combination of criteria, including degree of statistical significance, magnitude of differential gene expression, and possible biologic relevance to disease. Standard TaqMan assays (20 μL) were performed by using an Applied Biosystems Universal Assay Buffer with 10 ng of input cDNA per reaction and amplified for 40 cycles in an ABI Prism 7900HT Sequence Detection System (SDS software version 2.3). Co-amplication of human glyceraldehyde-3-phosphate dehydrogenase (GAPDH) served as an endogenous assay and standardization control. RQ of gene expression was calculated from log-phase mean threshold cycle values normalized to GAPDH expression by using Applied Biosystems RQ Manager software (version 1.2).

## Results

### Microarray differential expression analysis

Significant differences (FDR-adjusted *P *values of less than 0.1) were observed for 104 oligo probes in analyses of SAID probands compared with the unrelated, matched controls (Table 1). Upon probe annotation, 92 genes representing several non-mutually exclusive categories, including immune function (16%), signaling pathways (24%), transcription/translation regulators (26%), and metabolic functions (15%), were identified (Table 2). Of these 92 genes, 73.9% were underexpressed (mean fold change of 0.71 and range of 0.91 to 0.56) and 26.1% were overexpressed (mean fold change of 2.2 and range of 1.1 to 7.2) in probands relative to unrelated, matched controls.

**Table 1:** Genes that were differentially expressed between probands (*n *= 20) with systemic autoimmune disease and unrelated, matched controls (*n *= 40)

Accession	Annotation	Fold change^a^	*P *value^b^	FDR	Function/Category
**Increased expression in probands**					
*TNFAIP6*	Tumor necrosis factor-alpha-induced protein 6	2.2	1.5 × 10^-^^5^	0.051	Co-factor, immune
*HP*	Haptoglobin	2.2	2.0 × 10^-^^5^	0.051	Carrier, transport
*HPR*	Haptoglobin-related protein	2.3	3.5 × 10^-^^5^	0.059	Carrier, transport
*ANXA3*	Annexin A3	2.8	5.1 × 10^-^^5^	0.074	Regulation, signaling
*APOBEC3A*	Apolipoprotein B mRNA editing enzyme 3A	1.4	5.8 × 10^-^^5^	0.074	Regulation, translation
*EIF2AK2*	Eukaryotic translation initiation factor 2-alpha kinase 2	2.4	7.1 × 10^-^^5^	0.074	Regulation, translation
*BMX*	Non-receptor tyrosine kinase	2.2	8.6 × 10^-^^5^	0.074	Kinase, signaling
*CEACAM6*	Carcinoembryonic Ag-related cell adhesion molecule 6	4.7	8.7 × 10^-^^5^	0.074	Receptor, targeting
*PLSCR1*	Phospholipid scramblase 1	2.3	1.0 × 10^-^^4^	0.074	Regulation, immune
*LTF*	Lactotransferrin	2.3	1.1 × 10^-^^4^	0.074	Regulation, immune
*TNFSF10*	Tumor necrosis factor ligand 10	1.6	1.4 × 10^-^^4^	0.074	Cytokine, immune
*OASL*	2'-5'-Oligoadenylate synthetase-like	2.2	1.7 × 10^-^^4^	0.074	Binding, thyroid receptor
*MAP2K6*	Mitogen-activated protein kinase kinase 6	1.4	2.2 × 10^-^^4^	0.080	Kinase, signaling
*ZCCHC2*	Zinc finger protein	1.5	2.4 × 10^-^^4^	0.082	Unknown, signaling
*INHBB*	Inhibin, beta B	2.3	2.7 × 10^-^^4^	0.085	Ligand, signaling
*DEFA4*	Defensin, alpha 4, corticostatin	2.3	2.9 × 10^-^^4^	0.088	Ligand, signaling
*IFI27*	Interferon, alpha-inducible protein 27	7.2	3.0 × 10^-^^4^	0.088	Unknown, immune
*SLC22A4*	Solute carrier family 22, member 4	1.7	3.3 × 10^-^^4^	0.088	Transport
*IL1RN*	Interleukin 1 receptor antagonist	1.7	3.4 × 10^-^^4^	0.088	Ligand, immune
*RRAGD*	Ras-related GTP-binding protein D	1.3	3.4 × 10^-^^4^	0.088	Ligand, signaling
*DEGS1*	Degenerative spermatocyte homolog 1	1.1	3.9 × 10^-^^4^	0.093	Enzyme, metabolism
*PGAP1*	GPI deacylase	1.3	3.9 × 10^-^^4^	0.093	Enzyme, signaling
*F5*	Coagulation factor V	1.4	4.2 × 10^-^^4^	0.094	Ligand, coagulation
*CDS2*	CDP-diacylglycerol synthase	1.2	4.6 × 10^-^^4^	0.097	Enzyme, metabolism
*HIST1H2AB*	Histone 1, H2ab	1.3	4.7 × 10^-^^4^	0.097	Chromatin, structural
**Decreased expression in probands**					
*PACSIN1*	Protein kinase C substrate 1 in neurons	-1.4	3.3 × 10^-^^6^	0.042	Kinase, signaling
*KRTCAP2*	Keratinocyte-associated protein 2	-1.2	5.9 × 10^-^^6^	0.042	Enzyme, structural
*FCER1A*	Fc fragment of high affinity IgE receptor	-1.9	9.2 × 10^-^^6^	0.042	Receptor, immune
*FYN*	Oncogene	-1.3	9.8 × 10^-^^6^	0.042	Kinase, signaling
*SYTL2*	Synaptotagmin-like 2	-1.5	1.0 × 10^-^^5^	0.042	Unknown, immune
*CD99*	MIC2, single-chain type-1 glycoprotein	-1.2	1.8 × 10^-^^5^	0.051	Receptor, immune
*PECI*	Peroxisomal D3,D2-enoyl-CoA isomerase	-1.3	3.3 × 10^-^^5^	0.059	Enzyme, metabolism
*CD81*	Target of antiproliferative antibody 1	-1.2	3.4 × 10^-^^5^	0.059	Receptor, immune
*PBX4*	Pre-B-cell leukemia transcription factor 4	-1.4	7.7 × 10^-^^5^	0.074	Regulation, transcription
*DYRK2*	Tyrosine-phosphorylation regulated kinase 2	-1.2	8.1 × 10^-^^5^	0.074	Kinase, signaling
*ZBTB5*	Zinc finger and BTB domain containing 5	-1.2	9.4 × 10^-^^5^	0.074	Regulation, transcription
*PABPC3*	Poly(A)-binding protein 3	-1.3	9.8 × 10^-^^5^	0.074	Regulation, translation
*NELL2*	NEL-like 2	-1.3	1.0 × 10^-^^4^	0.074	Mitogen, cell division
*LOC100133315*	Unknown cation channel/receptor	-1.2	1.0 × 10^-^^4^	0.074	Receptor, signaling
*DNAJA3*	DnaJ (Hsp40) homolog A3	-1.3	1.2 × 10^-^^4^	0.074	Chaperone, transport
*EIF3S6IP*	Eukaryotic translation initiation factor 3, subunit 6	-1.3	1.2 × 10^-^^4^	0.074	Regulation, translation
*EIF3S8*	Eukaryotic translation initiation factor 3, subunit 8	-1.2	1.2 × 10^-^^4^	0.074	Regulation, translation
*RBMX*	RNA-binding motif protein	-1.1	1.2 × 10^-^^4^	0.074	Regulation, transcription
*GZMK*	Granzyme K	-1.4	1.3 × 10^-^^4^	0.074	Enzyme, immune
*DNMT1*	DNA (cytosine 5)-methyltransferase 1	-1.3	1.3 × 10^-^^4^	0.074	Enzyme, metabolism
*ZNF219*	Zinc finger protein 219	-1.2	1.3 × 10^-^^4^	0.074	Regulation, transcription
*DVL1*	Dishevelled homologue (Drosophila)	-1.2	1.4 × 10^-^^4^	0.074	Mitosis, signaling
*IMP3*	U3 small ribonucleoprotein homologue (yeast)	-1.2	1.5 × 10^-^^4^	0.074	Structural, translation
*ANAPC5*	Anaphase promoting complex subunit 5	-1.2	1.5 × 10^-^^4^	0.074	Enzyme, cell cycle
*AOF2*	Amine oxidase (flavin containing) domain 2	-1.2	1.6 × 10^-^^4^	0.074	Regulation, transcription
*GPRASP1*	G protein-coupled receptor	-1.4	1.6 × 10^-^^4^	0.074	Receptor, signaling
*LTK*	Leukocyte tyrosine kinase	-1.3	1.6 × 10^-^^4^	0.074	Kinase, immune
*MTA1*	Metastasis-associated 1	-1.2	1.6 × 10^-^^4^	0.074	Regulation, transcription
*UXT*	Ubiquitously expressed transcript	-1.2	1.6 × 10^-^^4^	0.074	Regulation, transcription
*KLRB1*	Killer cell lectin-like receptor B1	-1.6	1.7 × 10^-^^4^	0.074	Receptor, immune
*SEMA4C*	Semaphorin 4C	-1.3	1.7 × 10^-^^4^	0.074	Neural development
*STMN3*	Stathmin-like 3	-1.4	2.0 × 10^-^^4^	0.079	Cytoskeletal, signaling
*ATP1A1*	ATPase Na^+^/K^+ ^transporter	-1.2	2.0 × 10^-^^4^	0.079	Transporter, signaling
*PSBP*	Prostatic steroid-binding protein	-1.3	2.2 × 10^-^^4^	0.080	Receptor, signaling
*RPL18*	Ribosomal protein L18	-1.3	2.2 × 10^-^^4^	0.080	Structural, translation
*RNF220*	Ring finger protein	-1.1	2.2 × 10^-^^4^	0.080	Unknown
*SPOCK2*	Sparc/osteonectin/proteoglycan (testican) 2	-1.2	2.3 × 10^-^^4^	0.081	Extracellular, structural
*DDX28*	DEAD box polypeptide	-1.2	2.3 × 10^-^^4^	0.081	RNA helicase, transcription
*SRP46*	Splicing factor, arginine/serine-rich, 46 kDa	-1.3	2.4 × 10^-^^4^	0.082	Regulation, transcription
*EIF3S7*	Eukaryotic translation initiation factor 3S7	-1.2	2.5 × 10^-^^4^	0.084	Regulation, translation
*TNFRSF25*	Tumor necrosis factor receptor 25	-1.3	2.6 × 10^-^^4^	0.085	Receptor, immune
*MYBL1*	v-Myb myeloblastosis viral oncogene homolog	-1.4	2.7 × 10^-^^4^	0.085	Regulation, transcription
*UBE2I*	Ubiquitin-conjugating enzyme E2I	-1.1	2.8 × 10^-^^4^	0.087	Enzyme, regulation
*PABPC1*	Poly(A)-binding protein	-1.3	3.0 × 10^-^^4^	0.088	Regulation, translation
*LDOC1L*	Leucine zipper, downregulated in cancer 1-like	-1.4	3.1 × 10^-^^4^	0.088	Unknown
*LGR6*	Leucine-rich repeat-containing G protein receptor 6	-1.6	3.1 × 10^-^^4^	0.088	Receptor, signaling
*AES*	Amino-terminal enhancer of split	-1.1	3.2 × 10^-^^4^	0.088	Regulation, transcription
*CXCR3*	Chemokine receptor 3	-1.3	3.2 × 10^-^^4^	0.088	Receptor, immune
*ATP5G2*	ATP synthase, mitochondrial F0 complex	-1.2	3.3 × 10^-^^4^	0.088	Enzyme, metabolism
*RPLP2*	Ribosomal protein P2	-1.3	3.3 × 10^-^^4^	0.088	Structural, translation
*TC2N*	Membrane targeting C2 domain	-1.4	3.6 × 10^-^^4^	0.090	Unknown, signaling
*ERGIC3*	Breast cancer antigen 84	-1.2	3.8 × 10^-^^4^	0.092	Unknown, cell growth
*GPR183*	Epstein-Barr virus induced gene 2	-1.5	3.9 × 10^-^^4^	0.093	Receptor, signaling
*CSNK1E*	Casein kinase 1, epsilon	-1.2	4.1 × 10^-^^4^	0.094	Kinase, regulation
*CRIP1*	Cysteine-rich protein 1	-1.3	4.1 × 10^-^^4^	0.094	Zn-binding, transport
*ARIH2*	Ariadne homolog 2	-1.1	4.2 × 10^-^^4^	0.094	Ubiquitin ligase, regulation
*POLS*	DNA polymerase sigma	-1.2	4.2 × 10^-^^4^	0.094	Enzyme, metabolism
*PUF60*	Ro-RNP-binding protein	-1.2	4.3 × 10^-^^4^	0.096	RNP-binding, regulation
*MFNG*	Fucose-specific β-1,3-N-acetylglucosaminyltransferase	-1.2	4.7 × 10^-^^4^	0.097	Enzyme, development
*PRPSAP2*	Phosphoribosyl pyrophosphate synthetase protein 2	-1.2	4.7 × 10^-^^4^	0.097	Enzyme, metabolism
*STAG3*	Stromal antigen 3	-1.3	4.7 × 10^-^^4^	0.097	Cohesion subunit, meiosis
*FBL*	Fibrillarin	-1.2	4.9 × 10^-^^4^	0.098	snRNP-binding, pre-rRNA
*RPS19*	Ribosomal protein S19	-1.3	4.9 × 10^-^^4^	0.098	Structural, translation
*DDB1*	Damage-specific DNA-binding protein 1	-1.2	5.0 × 10^-^^4^	0.098	DNA repair
*FBXO21*	F-box protein 21	-1.2	5.0 × 10^-^^4^	0.098	Ubiquitin ligase, regulation
*PTDSS1*	Phosphatidylserine synthase 1	-1.2	5.1 × 10^-^^4^	0.100	Enzyme, metabolism
*AKR1B1*	Aldo-keto reductase family 1, member B1	-1.2	5.2 × 10^-^^4^	0.100	Enzyme, metabolism

^a^Fold change values indicate increased (positive value) or decreased (negative value) levels of gene expression in probands relative to unrelated, matched controls. Oligo probes for hypothetical or unidentified genes were omitted. ^b^Uncorrected. FDR, false discovery rate.

**Table 2:** Functional categorization of genes differentially expressed between SAID probands and unrelated, matched controls

Immune	Signaling	Gene expression	Metabolism	Transport	Structural	Cell cycle	Other/Unknown
*TNFAIP6*	*ANXA3*	*EIF2AK2*	*DEGS1*	*HP*	*HIST1H2AB*	*NELL2*	*ZCCHC2*
*PLSCR1*	*APOBEC3A*	*PBX4*	*CDS2*	*HPR*	*KRTCAP2*	*DVL1*	*RNF220*
*LTF*	*BMX*	*ZBTB5*	*PECI*	*SLC22A4*	*SPOCK2*	*ANAPC5*	*LDOC1L*
*TNFSF10*	*CEACAM6*	*PABPC3*	*DNMT1*	*DNAJA3*		*SEMA4C*	*DDB1*
*IFI27*	*OASL*	*EIF3S6IP*	*ATP5G2*	*CRIP1*		*ERGIC3*	
*IL1RN*	*MAP2K6*	*EIF3S8*	*POLS*			*STAG3*	
*FCER1A*	*INHBB*	*RBMX*	*MFNG*				
*SYTL2*	*DEFA4*	*ZNF219*	*PRPSAP2*				
*CD99*	*RRAGD*	*IMP3*	*PTDSS1*				
*CD81*	*PGAP1*	*AOF2*	*AKR1B1*				
*GZMK*	*PACSIN1*	*MTA1*					
*LTK*	*FYN*	*UXT*					
*KLRB1*	*DYRK2*	*RPL18*					
*TNFRSF25*	*LOC100133315*	*DDX28*					
*CXCR3*	*GPRASP1*	*SRP46*					
	*STMN3*	*EIF3S7*					
	*ATP1A1*	*MYBL1*					
	*PSBP*	*PABPC1*					
	*UBE2I*	*AES*					
	*LGR6*	*RPLP2*					
	*TC2N*	*FBL*					
	*GPR183*	*RPS19*					
	*CSNK1E*						
	*ARIH2*						
	*PUF60*						
	*FBXO21*						
	*F5*						

SAID, systemic autoimmune disease.

Comparisons between probands with SAID and their unaffected twins produced no significant differences in gene expression values (that is, the smallest adjusted *P *values were greater than 0.1), although several of the top-ranking genes were shared with those identified as significant in comparisons of probands and unrelated, matched controls. Comparisons of unaffected twins and unrelated, matched controls likewise revealed no significant differences in gene expression.

**Figure 1:**
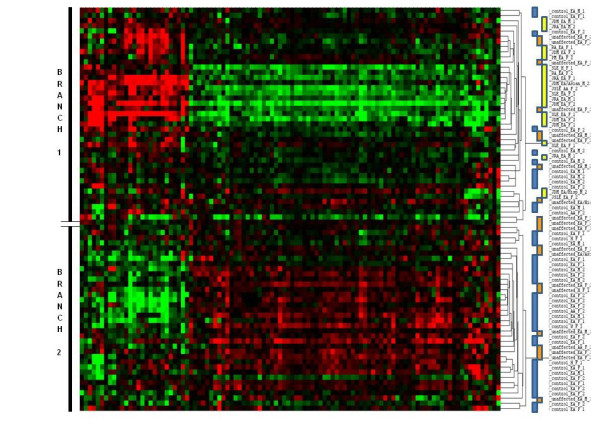
**Heat map representing an unsupervised, hierarchical cluster analysis of the three study groups**. The groups consisted of 20 probands with a systemic autoimmune disease, their 20 unaffected twins, and 40 unrelated, matched controls, respectively. The heat map uses 104 oligo probes that exhibited statistically significant differential gene expression (multiple comparison-adjusted *P *values (false discovery rate) of less than 0.1) between probands and unrelated, matched controls. Color codes to the immediate right of the dendrogram correspond to probands (yellow), unaffected twins (orange), and unrelated, matched controls (blue). The first major partition in the dendrogram (branches 1 and 2) is marked to the left of the heat map.

We conducted an unsupervised, hierarchical cluster analysis for all 80 subjects (20 probands, 20 unaffected twins, and 40 unrelated, matched controls) by using the expression data for the 104 probes with significant differential expression on the basis of the comparison of probands versus unrelated controls. The resulting heat map (Figure 1) demonstrated a clear segregation of the probands with SAID and the unrelated, matched controls. In contrast, the unaffected twins were interspersed among and between the probands and unrelated controls. The first two major branches of the dendrogram divided the heat map into approximately equal numbers of study subjects (that is, 40 in the top half and 40 in the bottom half of the heat map). While all probands segregate into the top half of the map (that is, Figure 1, branch 1), the unaffected twins were equally distributed between the two major branches. The unrelated, matched controls were distributed between the two major branches (approximately 1/3 in branch 1 and 2/3 in branch 2). We also observed that several probands mapped outside the primary proband cluster (branch 1). Further analyses of proband outliers did not reveal any significant differences in age, medications, disease duration, physical exam findings, patient- and physician-assessed levels of disease activity, white blood or platelet counts, or hematocrit when compared with probands in the primary cluster (data not shown).

**Figure 2:**
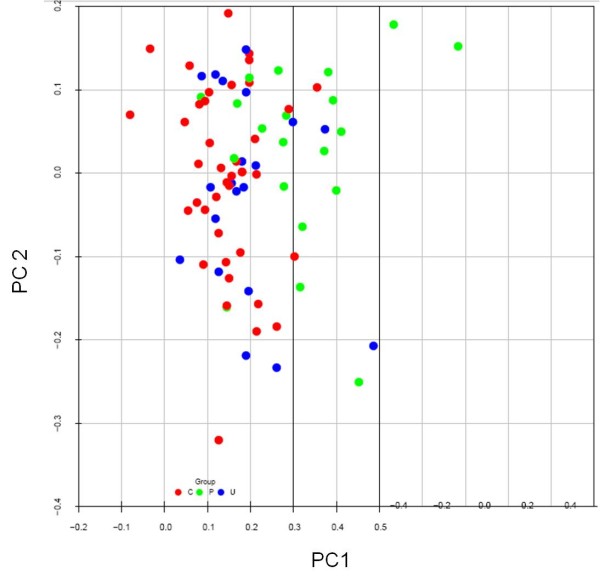
**Principal component analysis of the three study groups**. The groups consisted of 20 probands with a systemic autoimmune disease (green), their 20 unaffected twins (blue), or 40 unrelated, matched controls (red), respectively. The analysis uses 104 oligo probes that exhibited statistically significant differential gene expression (multiple comparison-adjusted *P *values (false discovery rate) of less than 0.1) between probands and unrelated, matched controls. C, unrelated, matched control; P, proband; PC, principal component; U, unaffected twin.

A PCA of the 80 study subjects by using the same 104 differentially expressed probes again revealed segregation of probands from unaffected twins and unrelated, matched controls (Figure 2). No differences due to age, gender, ethnicity, disease status, or treatment were observed across the first three principal components.

While the distinction between unaffected twins and unrelated, matched controls was less obvious in this PCA, our inability to detect any significant differences in gene expression between unaffected twins and either probands or unrelated, matched controls suggested that the gene expression profile of unaffected twins may signify an intermediate or transitional state between the healthy and disease phenotypes. We observed further that the average gene expression for unaffected twins was intermediate between that of twins with SAID and unrelated, matched controls for 6,535 (32%) of a total of approximately 20,000 probes on the chip (about one third of all probes might be predicted to show this pattern by chance alone). By contrast, the same intermediate ordering was observed for 84 out of 104 probes (81%) that were significantly different between probands and controls.

### Disease subtypes

By performing an unsupervised hierarchical clustering of all proband samples, we further examined whether probands might also segregate by disease subtype (that is, SLE, RA, and IIM). The clustering did not segregate probands into groups dominated by SAID subtypes (data not shown). Second, to identify probes that were significantly different among the disease groups, for each probe and each twin pair we calculated differences in log-transformed gene expression levels between unaffected and affected twins and compared these differences among disease groups with ANOVA. After adjustments for multiple testing, we did not detect any probes with significant *P *values among twin pairs whose probands had different diagnoses (data not shown).

To further assess these findings, we employed a mixed-effects model comparing probe expression values from probands and unrelated, matched controls, stratified by including a random effect for the matched sets, and comparing across disease phenotypes. Again, after adjustments for multiple testing, no significant differences in the change in gene expression (that is, the fixed-effects interaction between disease status and phenotype category) were observed. Together, these data suggest that either our disease-specific sample sizes were too small to detect significant differences or patients with different SAIDs possess similar gene expression patterns.

### Pathway analysis

**Figure 3:**
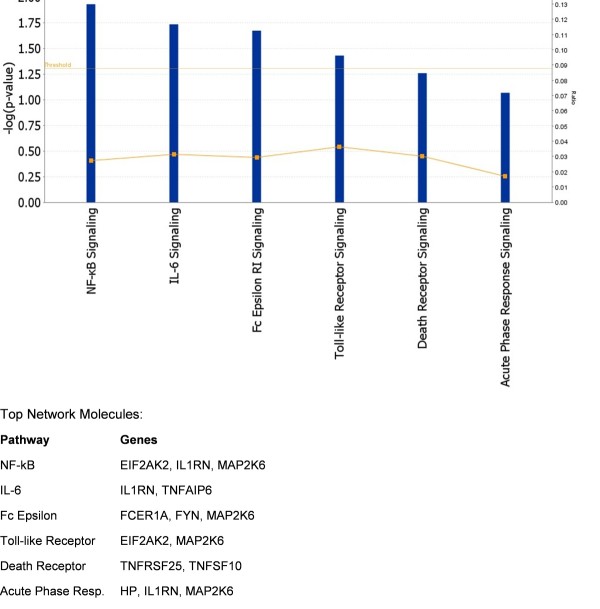
**Ingenuity pathways analysis of 104 probes with false discovery rate-adjusted *P *values of less than 0.1**. The analysis compares 20 twins affected with systemic autoimmune disease and 40 unrelated, matched controls. *P *values (blue bars) describing the confidence of the association of the data set with a given pathway are shown on the left vertical axis (-log(*P *value)). The cutoff threshold value (defined as *P *= 0.05) is also shown (horizontal yellow line). The ratio of the number of genes from the data set that map to a given pathway divided by the total number of molecules that comprise the pathway (orange line connecting blue bars) is shown on the right vertical axis. IL-6, interleukin-6; NF-κB, nuclear factor-kappa-B.

To examine the possible functional clustering of genes that were differentially expressed in SAID, we performed IPAs to assess whether differentially expressed genes might be linked by common biologic pathways. As shown in Figure 3, those findings having the greatest significance (that is, lowest *P *values) included several canonical biologic pathways involved in immune response and inflammation (signaling molecules for NF-κB, IL-6, FcεRI, Toll-like receptor signaling, apoptotic, and acute-phase responses). In some instances, genes expressed differentially in SAID were found to play a regulatory role in multiple immune response and inflammatory pathways. For example, mitogen-activated protein kinase kinase 6 (*MAP2K6*) plays a regulatory role in NF-κB, IL-6, FcεRI, Toll-like receptor, and acute-phase response pathways. Similarly, the interleukin 1 receptor antagonist (*IL1RN*) influences the NF-κB, IL-6, and acute-phase response pathways, and eukaryotic translation initiation factor 2-alpha kinase 2 (*EIF2AK2*) is likewise involved in NF-κB and Toll-like receptor signaling. These results suggest that alterations in expression of a limited number of genes may, in turn, influence the dysregulation of numerous integrated immune response and inflammatory pathways that are common to several SAIDs.

### RQ-PCR validation

RQ of differential gene expression among disease-discordant twin pairs and unrelated, matched controls was independently evaluated by RT-PCR analyses for several genes of interest (*KRTCAP2*, *MAP2K6*, *IL1RN*, *IFI27*, *SYTL2*, *FYN*, *LTK*, *EIF2AK2*, *FCERA1*, *LGR6*, *DEFA4*, *ANXA3*, *TNFAIP6*, *CEACAM6*, and *TNFSF10*) as detailed in Materials and methods. As summarized in Table 3, 13 of the 15 genes evaluated by RQ-PCR showed the same trend (that is, increased or decreased levels of gene expression) as those observed from the microarray analysis in comparisons of twin probands and unrelated, matched controls. Moreover, the observed magnitude of differential expression for these genes was generally similar for these independent microarray and RQ-PCR assays. An opposite trend of differential expression was observed for the *FYN *gene by these assays, although the magnitudes of differential gene expression were small (approximately 1.2-fold). For the *LTK *gene, RQ-PCR did not detect a difference in gene expression between probands and unrelated, matched controls whereas microarray analysis estimated a 2.3-fold increase in gene expression. Similar comparative analyses of unaffected twins demonstrated intermediate levels of differential gene expression versus unrelated, matched controls for the majority of genes surveyed.

**Table 3:** Comparison of differential gene expression values determined by relative quantitative-polymerase chain reaction and microarray analyses

Gene	RQ-PCR^a^		Microarray^a^	
	
	Affected	Unaffected	Affected	Unaffected
*TNFAIP6*	3.04	2.17	2.24	2.15
*TNFSF10*	1.50	1.37	1.58	1.33
*MAP2K6*	1.20	1.20	1.38	1.21
*IL1RN*	1.37	1.21	1.67	1.17
*IFI27*	1.20	2.01	7.24	6.21
*ANXA3*	3.18	1.82	2.79	2.43
*CEACAM6*	5.70	1.15	4.70	1.10
*DEFA4*	7.01	1.47	2.32	1.18
*EIF2AK2*	2.20	1.12	2.36	1.70
*FCERA1*	-1.82	-1.48	-1.76	-1.22
*SYTL2*	-1.93	-1.61	-1.49	-1.10
*LGR6*	-1.25	-1.13	-1.64	-1.11
*KRTCAP2*	-1.20	-1.20	-1.16	-1.07
*LTK*	1.02	1.21	2.32	1.06
*FYN*	1.23	1.19	-1.26	-1.01

^a^Fold change values indicating increased (positive value) or decreased (negative value) levels of gene expression in systemic autoimmune disease-affected or -unaffected twins compared with unrelated, matched controls. RQ-PCR, relative quantitative-polymerase chain reaction.

## Discussion

Pathology in human autoimmune diseases is thought to result from a complex interplay of multiple, often polymorphic, genetic loci and accumulating environmental exposures. The importance of genetic predisposition is evidenced by high disease concordance rates and genetic linkage analyses among family members [23,24]. Further evidence for the role of genetics in SAID comes from the identification of candidate susceptibility loci, especially genes encoding polymorphic variants of HLA class II antigen-presenting molecules [25]. These findings are supported by single-nucleotide polymorphism-based genome-wide association studies (GWASs) identifying large numbers of putative susceptibility markers positioned along multiple chromosomes [26]. Many of the confirmed GWAS disease markers map in close proximity to or within genes controlling immune response signaling pathways (*PTPN22*, *TNFAIP3*, *TRAF1*, *CD40*, and *REL*), transcriptional activation (*STAT4 *and *IRF5*), and cytokine production (*IL-12 *and *IL-23*) [26,27]. Interestingly, the majority of these disease susceptibility loci individually confer only modest disease risks (relative risk values of 1.2 to 1.5) [28]. Moreover, many genetic risk factors are shared among multiple autoimmune diseases (for example, SLE, RA, type 1 diabetes, and multiple sclerosis) and across different ethnic backgrounds, suggesting that the dysregulation of fundamental immune regulatory pathways underlies shared pathogenetic mechanisms [2,4,29].

We used RNA microarrays to measure differential gene expression among MZ twins discordant for SAID and unrelated, healthy controls matched on age, gender, and ethnicity. Gene expression levels may be influenced by subjects' genetic background, age, gender, and environmental exposures as well as by many experimental variables relating to clinical sampling, processing, and data analysis. Despite these potential confounders, an array-based study of peripheral blood cells from MZ twins demonstrated significantly less variation in the proportion of differentially expressed genes among twins affected with an SAID (<2%) compared with unrelated controls (>14%) [1]. Interestingly, MZ twin-derived lymphoblastoid cell lines exhibit near identical patterns of monoallelic expression from otherwise heterozygous, polymorphic loci, suggesting, at least in part, a role for shared genetic controls of transcription or genetic imprinting or both [30]. Unexpectedly, we did not detect any significant differences in gene expression among disease-discordant MZ twins in our study after correction for multiple comparisons. Similar profiles of gene expression were described for array analyses examining eight pairs of MZ twins discordant for multiple sclerosis, in which investigators found only a single gene, *IFN *alpha-inducible protein GIP3, differentially expressed in over half of the eight twin pairs [5]. In contrast, larger numbers of differentially expressed genes were identified from lymphoblastoid cell lines derived from 11 MZ twin pairs discordant for RA, although this analysis was not adjusted for multiple comparisons [31]. Curiously, similar patterns of differential gene expression have been observed among SAID patients and their healthy, first-degree relatives [2,23]. Collectively, these data support a role for both genetic and other non-genetic variables, including perhaps environmental exposures influencing disease development. Also, small changes in levels of differential gene expression (generally 1.2- to 2.5-fold variations) may not be statistically observable in studies of smaller numbers of twin pairs, including our present study.

For 84 out of 104 probes (81%) with statistically significant differential expression between twins with an SAID and unrelated, matched controls, we observed that gene expression values of unaffected twins are positioned between affected twins and unrelated controls, suggesting that unaffected twins may represent an intermediate or transitional state of disease development. Together, our results and previous studies of MZ twins argue for the importance of shared genetic determinants in regulating gene expression profiles. However, additional factors, including epigenetic modifications that accumulate in an age-dependent fashion (that is, epigenetic drift) as well as accumulating environmental exposures, may also influence asymmetric patterns of disease development among MZ twins [32,33]. Differential patterns of gene expression between the affected probands and unrelated controls could reflect either acquired modifications that were present before the onset of disease and could (to some extent) have conferred susceptibility to developing an SAID or phenotypic features of the syndrome itself, having less to do with disease etiology.

Heterogeneous phenotypes characterize the spectrum of clinical entities comprising SAID (for example, SLE, RA, and IIM). However, as a group, these diseases share many clinical, laboratory, genetic, autoantibody, and possibly pathogenetic features [34]. In fact, many patients present clinically with overlap syndromes sharing diagnostic criteria for multiple autoimmune diseases. Previous microarray studies of peripheral blood cells isolated from patients with four different autoimmune diagnoses (SLE, RA, multiple sclerosis, and insulin-dependent diabetes mellitus) were found to have similar profiles of differential gene expression relative to unrelated, healthy controls [4,6]. Moreover, a meta-analysis of gene expression array profiles demonstrated high concordance between SLE and RA, further emphasizing disease similarities [7]. Although our power to detect differences was limited, our data indirectly support these conclusions in that we were unable to detect significant differences in gene expression among SAID subtypes (six with (J)SLE, six with (J)RA, and eight with JIIM) by using three independent statistical analyses (ANOVA, mixed-effects models, and hierarchical clustering). Together, these data suggest that shared alterations in gene expression may underlie similar pathogenic mechanisms among different SAIDs.

Our findings have corroborated previous studies identifying upregulation of type I IFN response genes observed predominantly in SLE but also in RA and IIM [7,10,12,14,16,19,35,36]. In fact, altered expression of IFN-inducible genes has been correlated with disease activity in SLE and IIM (DM and polymyositis) patients undergoing therapy [13,17,36]. We likewise observed upregulation of several SLE-associated IFN response genes, including *OASL *(2.2-fold), *PLSCR1 *(2.3-fold), *EIF2AK2 *(2.4-fold), and *IFI27*, the last of which exhibited the highest degree of increased gene expression (7.2-fold) in our study. Moreover, our findings are consistent with a report of IFN response-associated gene expression in DM whereby factors *TNFAIP6*, *TNFSF10*, *OASF*, *PLSCR1*, *EIF2AK2*, and *IFI27 *were elevated in peripheral blood [16].

Our study has several limitations, including small sample sizes and limited statistical power, resulting from the challenges of identifying and recruiting qualified MZ twins discordant for SAID. Studies of MZ twins, however, help to mitigate confounding factors associated with genetic polymorphisms in studies of unrelated human subjects. Variations in peripheral blood cell composition, disease activity, and effects of immunosuppressive therapies may also present difficulties; however, statistical evaluations of these variables did not reveal evidence of effects on gene expression patterns. Methodological and other technical variables were evaluated in part by the use of independent RQ-PCR assays, which corroborated our microarray data for the majority of genes studied. Other limitations included the phenotypic heterogeneity of the SAIDs studied and variations in environmental exposure histories. Despite these variables, many of the differentially expressed genes and candidate molecular pathways identified in our study are consistent with findings reported in other studies of SAID [7,12-14,16,36]. Functional analysis of genes found differentially expressed in our SAID probands identified multiple immunoregulatory (NF-κB, IL-6, and apoptosis) and proinflammatory (acute-phase response and Toll-like receptor) pathways. Moreover, several of these genes (for example, *MAP2K6*, *EIF2AK2*, and *IL1RN*) have coordinated functions in the integration of multiple, non-mutually exclusive regulatory pathways that together may contribute to common features of the SAID phenotype. The functional implications of each of these pathways in autoimmune disease were reviewed recently [37].

## Conclusions

Gene expression profiling of biofluids and tissues from SAID patients and matched controls has provided a wealth of information into possible mechanisms of disease development, chronicity, and therapeutic response. Despite the variability of data anticipated with human clinical studies, some meaningful consensus has emerged [4,8]. As expected, we observed different gene expression profiles between disease probands and unrelated controls. Our identification of an SAID-associated profile of genes influencing multiple molecular pathways, including the immune response, cellular signaling, inflammation, transcriptional/translational, and other regulatory controls, is consistent with GWASs demonstrating large numbers of genetic susceptibility loci, each of which contributes only a modest degree of overall risk [26,28,29]. Moreover, the lack of statistically significant differences in gene expression detected between affected and unaffected twins emphasizes the importance of genetic susceptibility to disease whereby unaffected twins may represent a transitional state between health and disease. Our report of shared profiles of altered gene expression among SAID patients with different clinical phenotypes is consistent with the hypothesis that many SAIDs have common features of disease pathogenesis. The identification of relevant genes whose products regulate and integrate multiple physiologic pathways might permit the development of targeted therapeutics benefiting a broader spectrum of patients with multiple SAID phenotypes.

## Abbreviations

ANOVA: analysis of variance; C: unrelated, matched control; DM: dermatomyositis; FDR: false discovery rate; GAPDH: glyceraldehyde-3-phosphate dehydrogenase; GEO: Gene Expression Omnibus; GWAS: genome-wide association study; IFN: interferon; IIM: idiopathic inflammatory myopathy; IL-6: interleukin-6; IPA: ingenuity pathways analysis; JDM: juvenile dermatomyositis; JPM: juvenile polymyositis; JRA: juvenile rheumatoid arthritis; MZ: monozygotic; NF-κB: nuclear factor-kappa-B; NIH: National Institutes of Health; P: proband; PCA: principal component analysis; RA: rheumatoid arthritis; RQ: relative quantitation; RQ-PCR: relative quantitation polymerase chain reaction; RT-PCR: real-time polymerase chain reaction; SAID: systemic autoimmune disease; SLE: systemic lupus erythematosus; U: unaffected twin.

## Competing interests

The authors declare that they have no competing interests.

## Authors' contributions

TPO'H contributed to sample preparation, RQ-PCR studies, data and bioinformatic analyses, and manuscript preparation. LGR contributed to patient recruitment, clinical assessments and data analyses, and manuscript editing. LG contributed to sample preparation, RQ-PCR studies, data analysis, and manuscript editing. RF contributed to microarray analyses and data processing, bioinformatic analyses, and manuscript preparation and editing. RSP contributed to microarray analyses and data processing, bioinformatic analyses, and manuscript editing. DMU and CRW contributed to statistical analyses and manuscript editing. RRS contributed to data processing and analysis and to manuscript editing. DM contributed to data processing and analysis. MFG contributed to patient recruitment, clinical assessments, and manuscript editing. FWM contributed to study design, patient recruitment, clinical assessments, data analyses, and manuscript preparation and editing. All authors read and approved the final manuscript.
